# Provision and utilisation of health and nutrition services during COVID‐19 pandemic in urban Bangladesh

**DOI:** 10.1111/mcn.13218

**Published:** 2021-07-15

**Authors:** Phuong Hong Nguyen, Celeste Sununtnasuk, Anjali Pant, Lan Tran Mai, Shivani Kachwaha, Deborah Ash, Mohsin Ali, Santhia Ireen, Kristen Kappos, Jessica Escobar‐Alegria, Purnima Menon

**Affiliations:** ^1^ Poverty Health and Nutrition Division International Food Policy Research Institute Washington District of Columbia USA; ^2^ FHI Solutions Washington District of Columbia USA

**Keywords:** Bangladesh, COVID‐19, service delivery, service utilisation

## Abstract

The COVID‐19 pandemic is expected to have profound effects on healthcare systems, but little evidence exists on service provision, utilisation, or adaptations. This study aimed to (1) examine the changes to health and nutrition service delivery and utilisation in urban Bangladesh during and after enforcement of COVID‐19 restrictions and (2) identify adaptations and potential solutions to strengthen delivery and uptake. We conducted longitudinal surveys with health care providers (*n* = 45), pregnant women (*n* = 40), and mothers of children <2 years (*n* = 387) in February 2020 (in‐person) and September 2020 (by phone). We used Wilcoxon matched‐pairs signed‐rank tests to compare the changes before and during the pandemic. Services delivery for women and children which require proximity were severely affected; weight and height measurements fell by 20–29 percentage points (pp) for pregnant women and 37–57 pp for children, and child immunisations fell by 38 pp. Declines in service utilisation were large, including drops in facility visitations (35 pp among pregnant women and 67 pp among mothers), health and nutrition counselling (up to 73 pp), child weight measurements (50 pp), and immunisations (61 pp). The primary method of adaptation was provision of services over phone (37% for antenatal care services, 44%–49% for counselling). Despite adaptations to service provision, continued availability of routine maternal and child health services did not translate into service utilisation. Further investments are needed to provide timely and accurate information on COVID‐19 to the general public, improve COVID‐19 training and provide incentives for health care providers and ensure availability of personal protective equipment for providers and beneficiaries.

**Key messages**

- The COVID‐19 pandemic is expected to have profound effects on healthcare systems. The government of Bangladesh has concerns about diminished coverage and quality of maternal and child health services, but little evidence exists on service provision, utilisation, or adaptations.
- This study offers empirical investigations of maternal and child health and nutrition service provision from the supply side and service utilisation from the demand side before and during the COVID‐19 pandemic. We found that the COVID‐19 pandemic adversely affected health and nutrition service provision and utilisation for urban populations in Dhaka, Bangladesh despite mitigation efforts and adaptations.
- Further investments are needed to provide timely and accurate information on COVID‐19 to the general public, improve COVID‐19 training for health care providers, provide incentives for health care providers and ensure availability of personal protective equipment for both providers and beneficiaries.

## 1 INTRODUCTION

The COVID‐19 pandemic is expected to have profound, and potentially lasting, impacts on employment, public health, food security and poverty (ILO, 2020; Laborde et al., 2020; World Bank, 2020). The disruptive effects of the COVID‐19 pandemic and government response measures, such as lockdowns, may further negatively influence healthcare systems, with women and children among the most vulnerable to its consequences. Direct and indirect effects of the pandemic on maternal and child health could be devastating, and jeopardise the important gains made over the last several decades. Modelling estimates made early in the pandemic suggest that reductions in essential maternal and child health services, coupled with decreased access to food, could result in up to 1,157,000 additional child deaths and 56,700 maternal deaths (Roberton et al., 2020). These projected deaths could be greater, as the current models have not yet considered potential negative effects on maternal nutrition, micronutrient deficiencies or intrauterine growth (Akseer et al., 2020).

Managing the competing demands on health service providers and resources to combat COVID‐19 while ensuring the continuity and delivery of routine services is one of many challenges countries are facing. The World Health Organization (WHO) Pulse Survey reported that nearly all of the 105 countries surveyed across five regions experienced demand‐ and supply‐side disruptions to essential health services between March and June 2020 (WHO, 2020b). Half of surveyed countries reported staff redeployment to COVID‐19 relief measures, and 56% and 61% experienced partial or severe disruptions to antenatal care (ANC) services and routine immunisations at health facilities, respectively. Reductions in outpatient care attendance was reported by 76% of countries, mainly due to government lockdowns which hindered access to health services. This pulse survey, however, only represented the opinions of key informants with qualitative information on disruptions, highlighting the need for empirical evidence from community‐based health providers and beneficiaries to quantify the negative impact of COVID‐19 on maternal and child health and nutrition services.

Inadequate integration of maternal, infant and young child nutrition (MIYCN) services into routine healthcare service delivery was a concern in Bangladesh prior to the pandemic. A quality assessment of nutrition services provided during ANC in Bangladesh showed that delivery of services was poor: <30% of women received four key ANC nutrition services (weight measurement, provision of iron‐folic acid supplements, provision of calcium supplements, diet counselling), 25% of sick children had their weight checked against a growth‐chart, and <1% had their length/height measured (Billah et al., 2017). Previous national nutrition strategies sought to accelerate progress in reducing high rates of maternal and child undernutrition, but lacked coordination, had limited links with the healthcare system, and poor delivery of nutrition services (Billah et al., 2017). Furthermore, health policies have largely focused on delivery of health services and improvements in health and nutrition outcomes in rural areas despite increasing urbanisation (Govindaraj et al., 2018).

Several initiatives have been undertaken by the public and private sectors to deal with the healthcare system crisis brought on by COVID‐19. The Government of Bangladesh created a special insurance and stimulus package for health care providers and the Bangladesh Public Service Commission hired 2000 doctors and 5000 nurses to increase capacity at government facilities. Private entities are building hospitals with intensive care units, donating personal protective equipment (PPE), and providing cash support and food (Reza et al., 2020). Development partners including Alive & Thrive, UNICEF and the World Bank supported the National Nutrition Services to develop the national ‘Nutrition Essential Services Continuity Guidelines during COVID‐19 Pandemic’ (National Nutrition Services et al., 2020). The guidelines were officially approved and distributed in July 2020, specifying nutrition services to be delivered at different levels, service delivery modalities, and key messages for pregnant women, breastfeeding mothers, and caregivers of young children. The guidelines also included information on essential supplies and instructions for coordination with relevant platforms and sectors, monitoring and reporting, and transitioning from COVID‐19 to a “new normal” situation.

Despite these mitigation efforts, delivery and receipt of health and nutrition services face many challenges. Health providers are encountering competing demands required to execute COVID‐19 response duties while maintaining or re‐establishing the delivery of routine services, and are at increased risk for physical, emotional and psychological distress (Cabarkapa et al., 2020; Giannis et al., 2021; Hassan et al., 2021). Travel restrictions and reduced mobilisation could cause declines in the provision of maternal and child health services and the demand for them among clients across the health system. Statistics on routine monthly services show that the number of visits by pregnant and recently delivered women for maternal health services sharply declined across Bangladesh at the onset of COVID‐19 and the nationwide stay‐at‐home order (Ainul et al., 2020). These monitoring data provided a snapshot of health services such as ANC, facility deliveries, and postnatal care, but did not have in‐depth information on nutrition services and couselling, or challenges and adaptations.

This paper aimed to (1) quantify the effect of the COVID‐19 pandemic and COVID‐19 restrictions on health and nutrition service provision and utilisation by pregnant women and mothers of children under 2 years of age in Dhaka City, Bangladesh, and (2) identify adaptions and a range of feasible solutions that have the potential to strengthen delivery and uptake of essential health and nutrition interventions in the context of COVID‐19 and beyond.

## 2 METHODS

### 2.1 Study context

Before the COVID‐19 pandemic, the Alive & Thrive (A&T) initiative began implementing a programme of interventions designed to standardise the delivery of MIYCN counselling services in eight existing urban Maternal Neonatal and Child Health (MNCH) facilities. These facilities, run by the non‐governmental organisations Radda Maternal and Child Health and Family Planning Centre (Radda) and Marie Stopes Bangladesh (MSB), provide maternal and child health services in urban areas of Dhaka City. The baseline survey for the quasi‐experimental impact evaluation of the programme was conducted in February 2020, before the onset of the pandemic in Bangladesh (Nguyen et al., 2020). The survey used mixed data collection methods including in‐person client interviews, facility assessments, provider surveys and case observations of ANC visits and counselling sessions. The sampling frame for the baseline survey and the data collected provided a unique opportunity for a pre‐ and post‐assessment of the effects of the pandemic and disruptions to health and nutrition service delivery and utilisation in Dhaka City.

On 26 March 2020, roughly 2 weeks after the first case of COVID‐19 in the country was confirmed, the Government of Bangladesh issued a nationwide extended public holiday and strict stay‐at‐home order. Formal and informal economic activities were restricted, educational institutions were closed, and public transportation by road, rail and air was suspended to control community transmission of the virus. Health facilities continued to provide essential health services, but faced intermittent disruptions and closures. The initial stay‐at‐home order remained in place until 30 May 2020 after which localised restrictions were enforced.

This current study utilises the existing contacts of health providers, pregnant women, and mothers from the February 2020 baseline survey to conduct phone surveys in September–October 2020, aiming to evaluate the effects of the COVID‐19 pandemic on service delivery and receipt before, during and after the stay‐at‐home orders went into effect. Phone surveys were considered a safer means for data collection at follow‐up than in‐person surveys, due to travel restrictions and the high risk of transmission of COVID‐19. Both in‐person and phone survey data collection were conducted by the same research firm, Data Analysis and Technical Assistance (DATA).

### 2.2 Health provider survey

We conducted longitudinal quantitative surveys with health providers at eight health facilities operated by Radda and MSB. In February 2020, we undertook in‐person surveys with 59 health providers to assess their exposure to training on MIYCN, workload and time commitments, and services provided, especially health and nutrition services for mothers and young children. In September–October 2020, we followed up with the same health providers to collect information on trainings, receipt of personal protective equipment (PPE), additional responsibilities, service provision for pregnant women and mothers with children <2 years, challenges and adaptations for service delivery, and additional support needed during the COVID‐19 pandemic. For all service‐related questions, respondents were asked to recall services provided during March–May 2020 and in September 2020, the month preceeding the survey. Health providers were also asked about communication channels, supervision, and their knowledge of COVID‐19 symptoms and prevention.

### 2.3 Pregnant women and mother surveys

In‐person surveys with pregnant women (*n* = 498) and mothers with children <2 years (*n* = 528) who were clients at the Radda and MSB health facilities were conducted in February 2020 to assess their receipt of health and nutrition services. In September 2020, phone interviews were administered with the same respondents to understand how service utilisation had changed during March–May 2020 and in September 2020. Questionnaires for pregnant women and mothers included modules on exposure to health and nutrition services, counselling messages, mode of service receipt, and challenges faced in accessing services.

### 2.4 Data analysis

We conducted descriptive analysis to report characteristics of health providers, pregnant women and mothers. Changes in service provision and utilisation were calculated over three periods: (1) February–May 2020, between pre‐pandemic time and the COVID‐19 stay‐at‐home orders and restrictions, to assess service disruption; (2) May–September 2020, when restrictions were lifted in phases, to assess service resumption; and (3) February–September 2020, before the onset of the pandemic through the easing of COVID‐19 restrictions. Wilcoxon matched‐pairs signed‐rank test was used to determine whether the differences in means were statistically significant. All statistical analyses were carried out with Stata (StataCorp, version 16).

### 2.5 Ethical considerations

Verbal consent was obtained from pregnant women, mothers and health care providers prior to their participation in the study. The research protocol received ethical clearance from the Institutional Review Board at the International Food Policy Research Institute and the Bangladesh Medical Research Council. Additional permissions for data collection were provided by Radda and MSB.

## 3 RESULTS

### 3.1 Characteristics of the study sample

Of the 59 health providers interviewed in February 2020, 45 completed interviews via phone (a response rate of 76%). Among those who did not participate, seven health providers changed jobs or moved to other places, two refused to participate, and five were unavailable (Table S1). Half of the health providers were paramedics and nearly a quarter were physicians or medical officers, employed for an average of 5 years at the facility (Table S2). Two thirds had a college degree or higher education level.

Since the majority of pregnant women had already delivered at the time of follow up, only 80 pregnant women of the 498 surveyed in February 2020 were eligible to be interviewed and 40 completed the interview (a response rate of 50%). Among mothers with children under 2 years of age, 73% (*n* = 387) of the sample from February 2020 were interviewed. Of those who did not participate, 84 had their phones switched off, 34 did not answer the enumerators' calls or the wrong phone number was provided, and 18 refused to participate or continue with the interview (Table S1). Pregnant women were on average 23 years of age and in their 36.5 week of pregnancy (Table S2). Mothers were 25 years of age on average and the mean age of their young child was 10 months. Ninety percent of both groups of women were housewives and approximately two thirds of them had obtained middle school education or higher.

### 3.2 Resources and health provider knowledge to respond to COVID‐19

**FIGURE 1:**
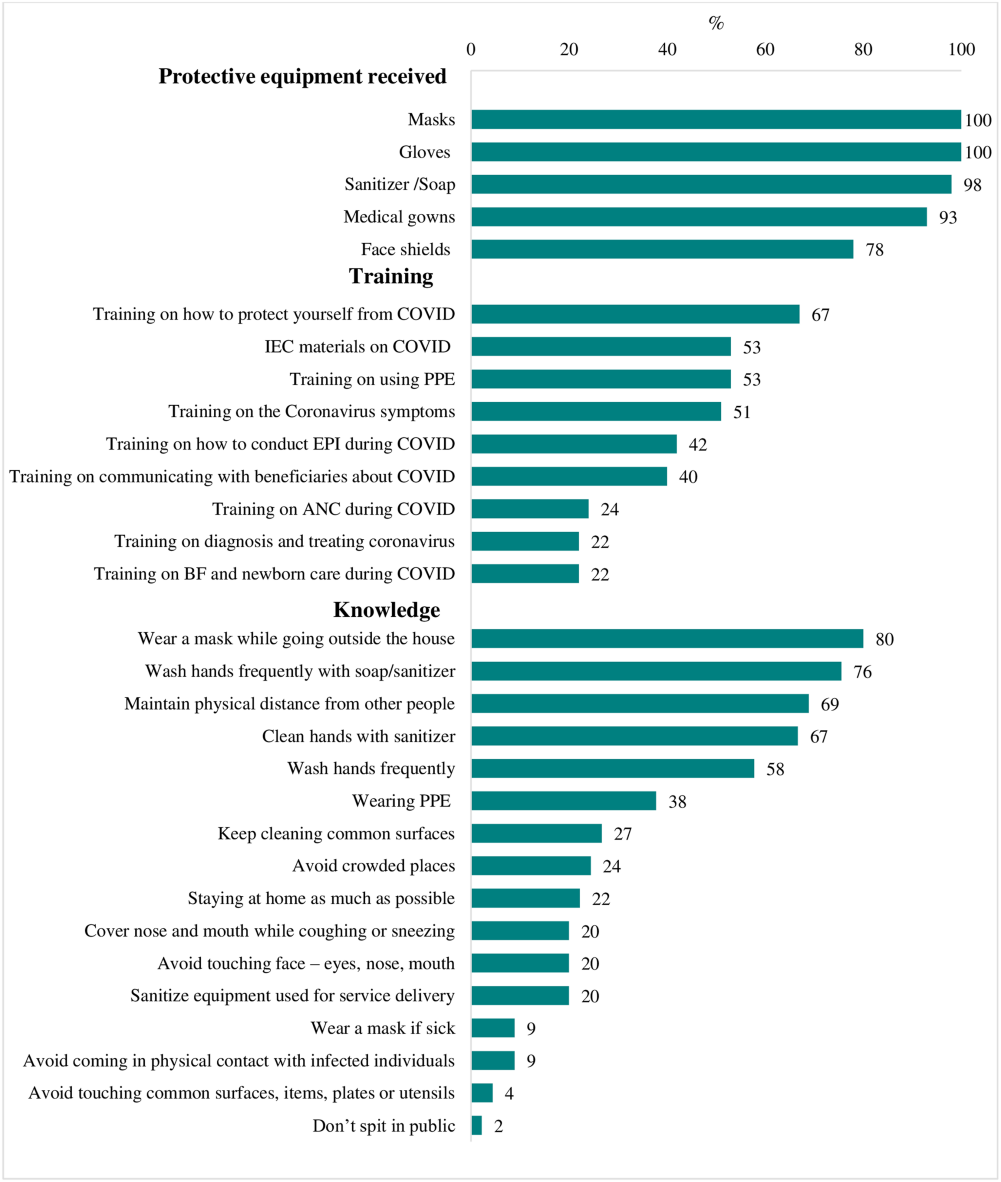
Resources for and knowledge of health providers to respond to COVID‐19^1^. ^1^Data from health providers' survey (*n* = 45). ANC: antenatal care; EPI: expanded program on immunisation; IEC: information, education and communication; PPE: personal protective equipment

Nearly all health providers were supplied with PPE, including masks, gloves, sanitiser/soap and medical gowns (Figure 1). Face shields were provided less frequently, but three out of four providers still received them. Trainings on COVID‐19 were less universally received. More than half of health providers received information, education, and communication materials on COVID‐19 or training on using PPE, how to protect oneself from COVID‐19 or on how to identify symptoms. But fewer than a quarter received training on how to adapt ANC services during COVID‐19 or provide breastfeeding or newborn care.

More than two thirds of providers knew to wash hands frequently, wear a mask when outside the home, and maintain physical distance from others. Roughly 40% linked PPE with transmission prevention and less than a quarter mentioned avoiding touching one's face, nose or mouth, or sanitising equipment used for service delivery.

### 3.3 Service provision before the pandemic (February 2020), during stay‐at home orders and travel restrictions (March–May 2020) and in the month preceding the survey (September 2020)

The impacts of the pandemic and COVID‐19 restrictions on service delivery are shown in Table 1. Compared to the pre‐pandemic period, fewer health facilities provided ANC services (falling by 6.6 pp) during the lockdown but health providers reported that most services for pregnant women during ANC continued to be offered. One exception was anthropometric measurements, which dropped by 20 pp for weight measurements and 29 pp for height measurements during the lockdown and continued to be at lower levels in September 2020 compared to the pre‐pandemic (by 14 and 28 pp, respectively). Prescription of and counselling on iron and folic‐acid (IFA) and calcium supplementation fell by less than 4 pp. Delivery of counselling on diet diversity, food intake, weight gain, and rest also decreased beyond the lockdown period, dropping by 4.2–12.5 pp by September 2020.

**TABLE 1:** Service delivery before the COVID‐19 pandemic, during the lockdown and in the previous month, in‐person survey December 2019 and phone survey August 2020^a^

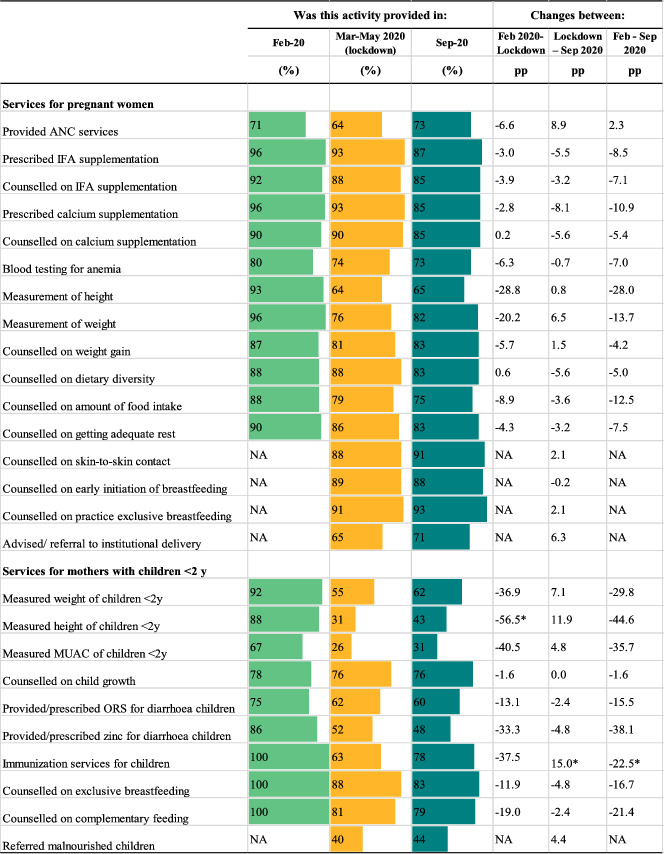

^a^ Data from health providers' survey (*n* = 45). The percentages of service delivery represent providers' reports of these services being available. ANC: antenatal care; IFA: iron and folic acid supplement; MUAC: mid‐upper arm circumference; NA: Not available; ORS: oral rehydration solution; pp: percentage point.

* *p* < 0.05.

Facility services provided for mothers with children under 2 years of age, particularly those which require proximity, were more severely affected (Table 1). During COVID‐19 restrictions, measurements of weight, height, and mid‐upper arm circumference to assess the child's growth dropped by 37, 57 and 41 pp, respectively, and child immunisation services fell by 38 pp. Counselling on exclusive breast feeding and complementary feeding fell by 12 and 19 pp. By September, some facilities resumed these services, though provision was still markedly below pre‐pandemic levels.

### 3.4 Service utilisation before the pandemic (February 2020), during stay‐at‐home orders and travel restrictions (March–May 2020) and in the month preceding the survey (September 2020)

Though the provision of most ANC services continued throughout the lockdown, service utilisation among pregnant women declined substantially, resulting in lower coverage of the target population. Facility visitations among pregnant women fell by 35 pp (Table 2). Counselling on IFA supplementation, which 80% of pregnant women reported receiving in February, fell by 73 pp. Significant declines in utilisation or receipt of other ANC services included measurement of weight (38 pp), receipt of IFA supplements (28 pp), and receipt of counselling on calcium supplementation (28 pp), diet diversity (25 pp), and quantity of food intake (35 pp). Health facility visitations continued to drop after the lockdown was lifted (8 pp) but other services increased modestly (between 5.0 and 15 pp) in September 2020.

**TABLE 2:** Services utilisation before the COVID‐19 pandemic, during the lockdown and in the previous month^a^, in‐person survey December 2019 and phone survey August 2020^a^

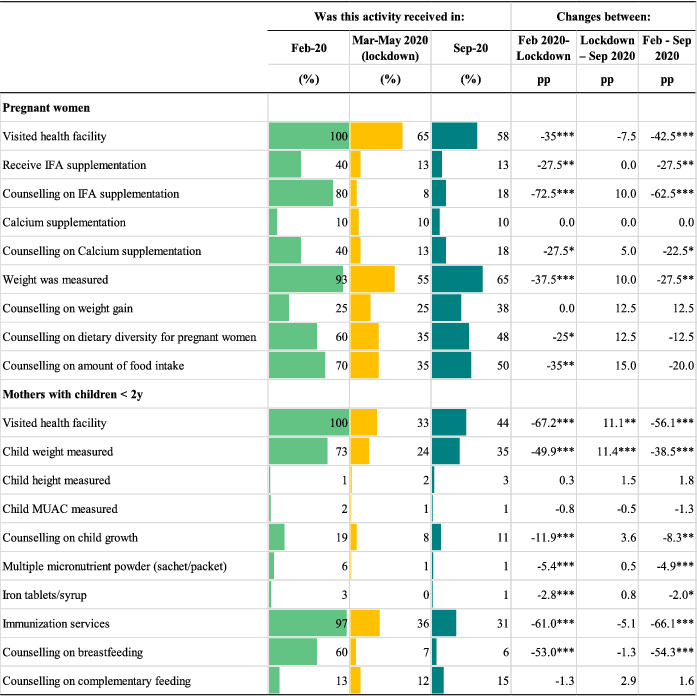

^a^ Data from survey with pregnant women (*n* = 40) and mothers of children <2 years (*n* = 387). The percentages of service utilisation represent beneficiaries' reports of these services being utilised. IFA: iron and folic acid supplement. MUAC: mid‐upper arm circumference.

* *p* < 0.05.

** *p* < 0.01.

*** *p* < 0.001.

Despite health facilities continuing to provide several services during the lockdown for mothers with children under 2 years of age, visitations for these services fell sharply (by 67 pp) (Table 2). Child weight measurements and counselling on breastfeeding dropped by 50 and 53 pp, respectively. Alarmingly, fewer women sought immunisation services for their children; utilisation fell by 61 pp during the lockdown and by additional 5.1 pp by September 2020. Facility visitations and child weight measurements increased by ~11 pp after COVID‐19 restrictions were lifted, but other service utilisation rates remained relatively the same through September 2020.

### 3.5 Challenges in service provision and utilisation

**FIGURE 2:**
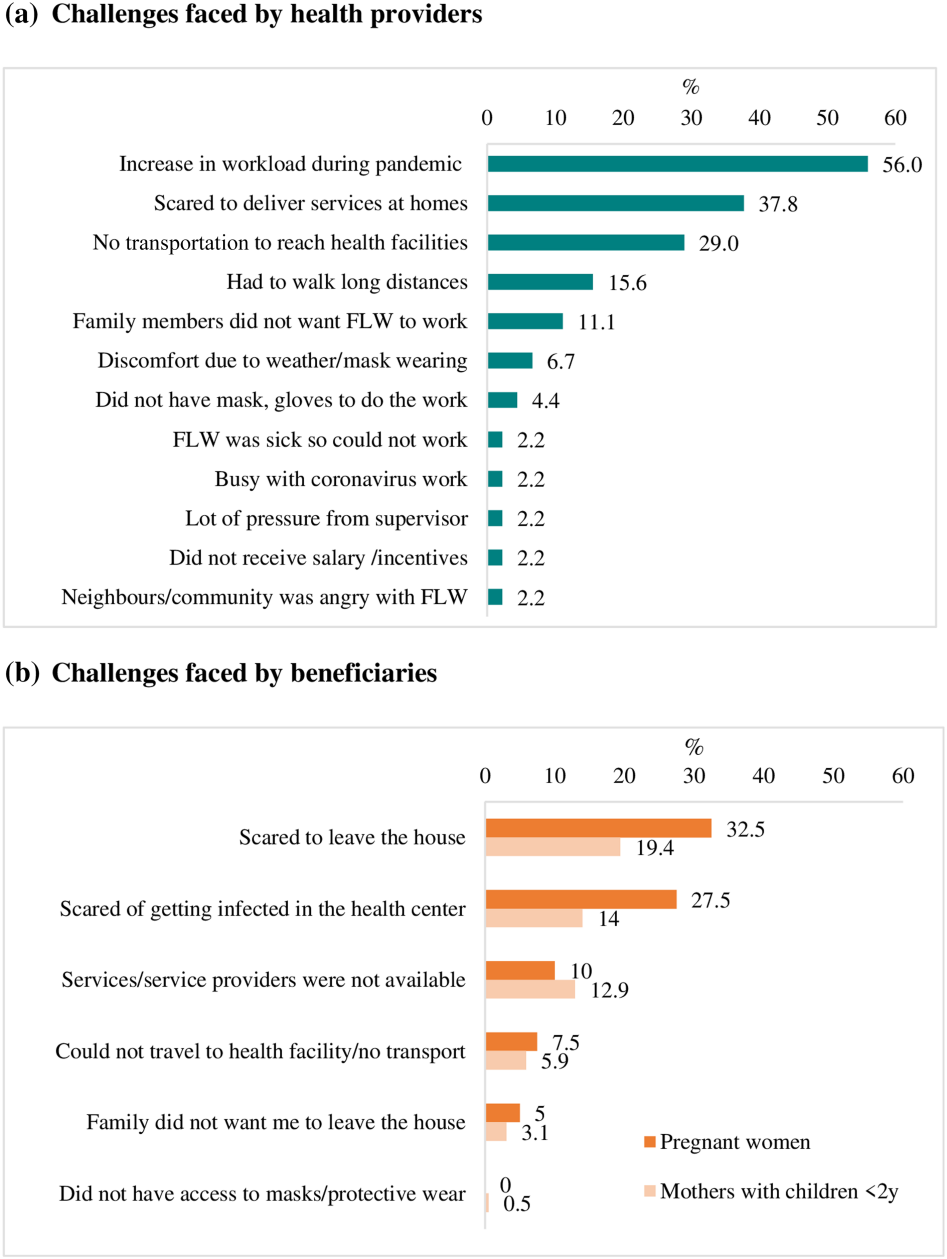
Challenges faced in providing and using services during the pandemic. (a) Challenges faced by health providers. (b) Challenges faced by beneficiaries. FLW: frontline workers

Increased workload during the pandemic was the most frequently mentioned difficulty faced by health providers (56%), followed by scared to deliver services at home (38%) and lack of transportation to reach heath facilities (29%) (Figure 2). Lack of adequate modes of transportation also meant that health providers had to walk long distances to their workplace (16%). Health providers' family fears of the perceived risks and prevented them continuing to work during the pandemic were also reported (11%).

The most frequently cited challenges for pregnant women and mothers to visit health facilities were fears of leaving the house (33% and 19%) and getting infected with COVID‐19 at the health centre (28% and 14%) (Figure 2). Unavailability of services or service providers was reported by roughly one in 10 women. Travel limitations due to lack of transportation or families wanting women to remain at home were faced less frequently (3%–8%).

### 3.6 Adaptations to service provisions

Health provider adaptations to routine methods of service delivery are presented in Table 3. Most health providers reported making adaptations to ANC services, and nearly all reported making adaptations to counselling services for pregnant women and mothers. The primary method of adaptation was provision of services over phone (37% for ANC services, 49% for counselling of pregnant women, and 44% for counselling of mothers with young children). More than half of health providers adapted services for child immunisations, including coordinating with their colleagues (48%) or supervisors (16%) to arrange immunisation drives, calling mothers to schedule immunisation appointments (36%), and sending reminders to mothers via WhatsApp messages or phone calls to encourage them to adhere to immunisation schedules (16%). Additional adaptations were also made on counselling content by adopting the ‘Nutrition Essential Services Continuity Guidelines during COVID‐19 Pandemic’ (National Nutrition Services et al., 2020) to be used during regular and mobile provision of services and supportive supervision, including messages on maternal nutrition and emphasising the protection, promotion, and support of appropriate and safe feeding for children during the COVID‐19 pandemic.

**TABLE 3:** Adaptation for services provision during the pandemic

	Services for pregnant women	Services for mothers with children <2 years
**Adaptation for ANC**		
Via phone	36.8	NA
Delivered to beneficiary homes	5.3	NA
Reminded through WhatsApp message/phone call	2.6	NA
Other	5.3	NA
**Adaptation for child immunisation**		
Coordinated with other colleagues to arrange appointments at immunisation drives	NA	48.0
Made an appointment for immunisation	NA	36.0
Reminded through WhatsApp message/phone call	NA	16.0
Coordinated with supervisor to arrange appointments at immunisation drives	NA	16.0
Arranged for transportation to immunisation drives	NA	4.0
Other	NA	8.0
**Adaptation for counselling**		
Using a phone call	48.8	43.9
At a community event	7.0	7.3
Using a video call	2.3	2.4
Counsel mothers on practices to continue breastfeeding during COVID‐19	NA	75.6

Abbreviations: ANC, antenatal care; NA, not available.

### 3.7 Additional support required for service provision

**FIGURE 3:**
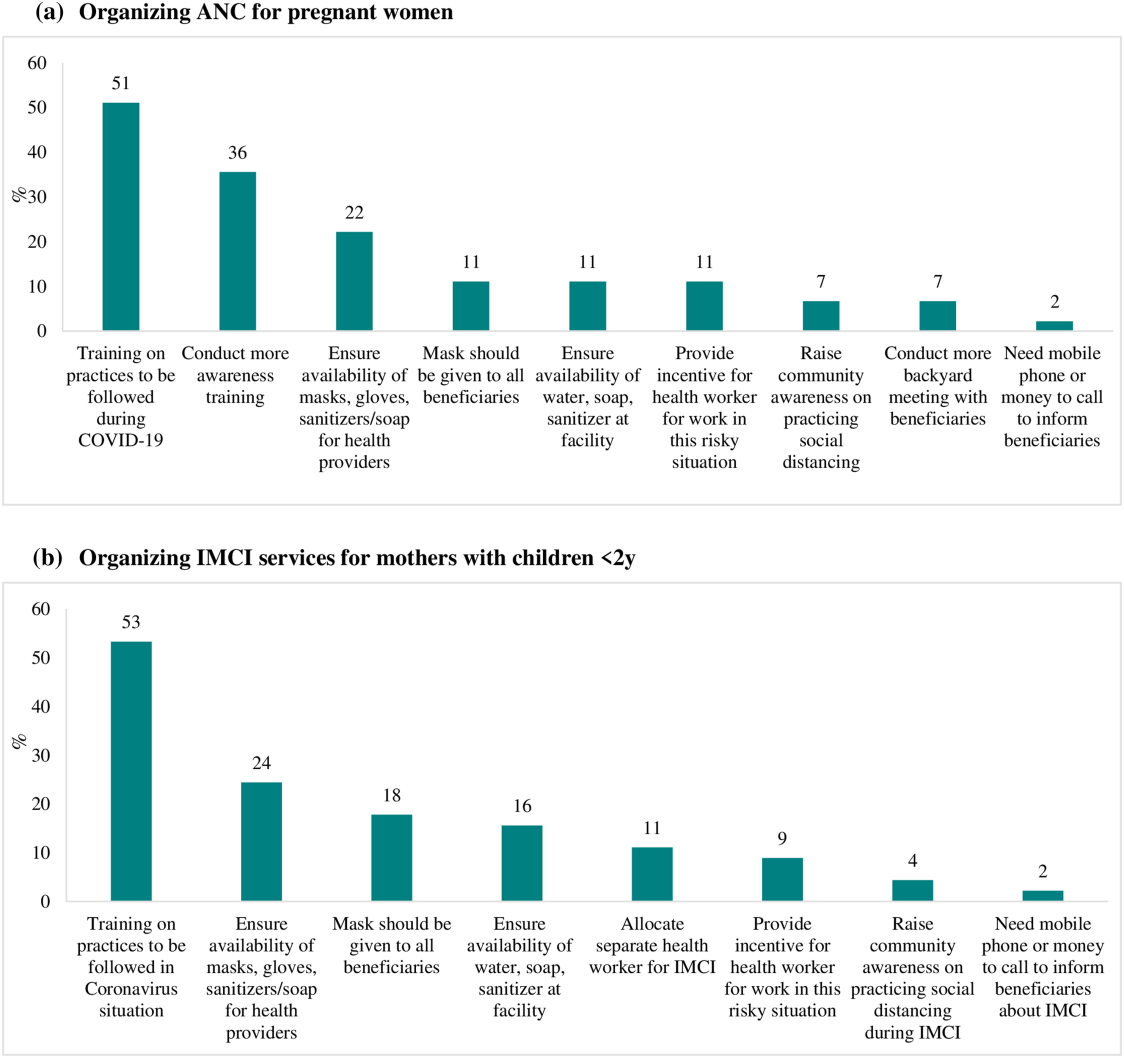
Additional resources or support needed by health providers for service provision. (a) Organising ANC for pregnant women. (b) Organising IMCI services for mothers with children <2 years. ANC: antenatal care; IMCI: integrated management of childhood illness

Additional resources and support needed by health care providers are presented in Figure 3. Training on best practices to follow during COVID‐19 (e.g., what services to provide and how to provide them) was the most cited need mentioned for those providing ANC services (51%) and services for mothers with young children (53%), with ANC providers further mentioning awareness trainings on safety protocols that workers should follow (36%). Providers of ANC and integrated management of childhood illness (IMCI) services listed ensured availability of masks, gloves, face shields and sanitisers/soaps for health providers (22% and 24%, respectively), provision of masks to all beneficiaries (11% and 18%), and availability of water, soap and sanitiser at the health facility (11% and 16%) as additional support needed. Incentives to work during the pandemic and offset the heightened risks was also mentioned by providers (9%–11%).

## 4 DISCUSSION

Our study is one of the first empirical investigations of urban MNCH service provision and utilisation before and during the COVID‐19 pandemic in Bangladesh. Using longitudinal surveys of health providers, pregnant women and mothers of children <2 years of age, we document the reported challenges and adaptations by providers and beneficiaries during the nationwide stay‐at‐home order and 4 months after restrictions were eased. Despite adaptations to service provisions, continued availability of routine maternal and child health services did not translate into service utilisation.

On the supply side, disruptions to services provided for pregnant women and mothers of young children were most pronounced among those requiring in‐person proximity, namely anthropometric measurements of pregnant women and children, and child immunisations. The provision of these services improved by September 2020 but remained markedly low compared to pre‐pandemic levels. Our results align and complement national monitoring data from the Bangladesh Ministry of Health and Family Welfare which showed severe declines in the provision of ANC and postnatal health services at government health facilities during COVID‐19 restrictions, with limited resumption of services in most districts after restrictions eased (Ainul et al., 2020), though the levels of decline were much smaller in our sample compared to national statistics. The National Nutrition Council of Bangladesh also used routine health system monitoring data to assess and anticipate the impacts of COVID‐19 on malnutrition cases via reduced access to services for pregnant and recently delivered women. Both the coverage and quality of services were found to have declined. During stay‐at‐home orders and travel restrictions, the number of ANC visits by mothers fell by 31%, and nutrition counselling and IFA distribution during ANC visits fell by 33% and 34%, respectively. While nutrition services for pregnant and lactating women became more available in June, they had still not returned to pre‐COVID‐19 levels by August (Bangladesh National Nutrition Council, 2020).

Government health facilities in Bangladesh are reported to have dealt with overcrowding, poor hygiene practices, lack of sanitation facilities, and acute shortages of PPE (Hassan et al., 2021; Reza et al., 2020), potentially exacerbating the inability of health providers to safely continue the provision of routine MCHN services. Standard training on COVID‐19 management and prevention have also largely been lacking for all types and levels of health workers in Bangladesh (Bangladesh National Nutrition Council, 2020). Higher levels of training and PPE in our study health facilities could be explained by our limited sample of urban‐NGO facilities, which may be better equipped and prepared to deliver services during the pandemic than government facilities. Nearly all providers reported receiving some PPE and roughly half received information, education, and communication materials on COVID‐19. Though suboptimal, our survey results indicate higher levels of provider preparedness for services during the pandemic compared to healthcare providers in other countries. A cross‐sectional survey of health professionals across 81 countries found only 30% received training and half received guidelines on care provision during COVID‐19 (Semaan et al., 2020). Among healthcare workers in Libya, only half of those surveyed received adequate training; they also had low awareness of and preparedness for COVID‐19 (Elhadi et al., 2020). Increased workloads, anxiety over risk of infection and, sometimes, harassment over stigma and opposition to the government's pandemic response measures have increased stress on health care providers. Fear of contracting COVID‐19 at health facilities was not unique to beneficiaries. Family members of health providers also expressed objections to their working during the pandemic (11%). The percentage of health providers facing this challenge may be underreported in our sample, as A&T records indicated high health facility staff turnover for this reason. Those staff who had already resigned by the time of survey are therefore not represented in the data.

On the demand side, service utilisation by pregnant women and mothers fell drastically during the pandemic. Fears of contracting the virus and hesitancy to leave their homes was reported by more than half of the women in our sample, potentially impacting their time spent in the clinic and ability to fully utilise the available in‐person services. Overall, health facility visits by pregnant women and mothers fell by 35 and 67 pp during the stay‐at‐home order, respectively, with visits falling an additional 7.5 pp among pregnant women 4 months after COVID‐19 restrictions were lifted. Precautionary measures and restrictions meant to slow the transmission of COVID‐19 forced health providers to adapt routine methods of service delivery. Yet, despite health providers adapting counselling services by switching to mobile phone for pregnant (49%) women and mothers of young children (44%), utilisation of counselling services among both groups of women fell. Large, significant drops were observed for IFA (63 pp) and food intake (20 pp) counselling for pregnant women and breastfeeding counselling (66 pp) for recently delivered women by September 2020. Continued low levels of utilisation among pregnant women and mothers of young children indicate an urgent need for motivating and supporting beneficiaries to continue seeking routine health services. Declines in family income and increases in food insecurity reported among rural Bangladeshi women and their families (Hamadani et al., 2020) may create additional financial barriers and constraints in accessing services, indicating further support needed for beneficiaries. Policy strategies to improve utilisation could include awareness campaigns, mass media, and social protection to support households suffering from the socio‐economic impacts of the pandemic.

To the best of our knowledge, literature on service provision and utilisation during COVID‐19 is limited, especially in low‐ and middle‐income countries. Widespread perceptions of reduced maternal care and essential health services have been qualitatively reported by health providers and health officials but have not been supported by empirical findings (Semaan et al., 2020; WHO, 2020a). We find similar reductions in MNCH service utilisation to those reported in a recent study conducted in Uttar Pradesh, India, where utilisation for most services failed to resume after frontline workers resumed service provision (Nguyen et al., 2021). Our results are also consistent with reports from the Government of Bangladesh that health facility closures, travel restrictions, fear of transmission, lack of confidence in the health system, and mental stress contributed to reductions in health service utilisation despite improvements in coverage and quality once restrictions lifted (Bangladesh National Nutrition Council, 2020).

This study offers a unique perspective on service provision from supply side and service utilisation from demand side for urban population, which is very limited till date. Using longitudinal samples of both health providers and beneficiaries, we provided insight information at three key time points: Before the onset of the pandemic, during government enforcement of COVID‐19 restrictions, and after restriction was lifted. Our results align and complement relevant data from the Government of Bangladesh (Bangladesh National Nutrition Council, 2020) and advance our understanding of the effects of COVID‐19 lockdown on the provision, access and uptake of health and nutrition services and of solutions to strengthen essential interventions in the context of COVID‐19 and beyond.

Bearing similar challenges as other phone surveys (Kempf & Remington, 2007), the response rate was lower among mothers in our phone survey (76%). The sample size of pregnant women at follow‐up was small, given that most women had already delivered by the time of the survey. Although we cover a primary form of private health service provision in urban areas, our sample is not representative of the universe of urban health providers and clients of these facilities. Finally, to reduce the phone survey length and complexity, questions capturing adaptations to maternal and child services mainly focused on delivery rather than counselling content.

## 5 CONCLUSION

The COVID‐19 pandemic adversely affected health and nutrition service provision and utilisation for urban populations in Dhaka, Bangladesh, despite mitigation efforts and adaptations. Further investments are needed to provide timely and accurate information on COVID‐19 to the general public, improve COVID‐19 training for health care providers, provide incentives for health care providers, and ensure availability of PPE for both providers and beneficiaries.

## CONFLICTS OF INTEREST

The authors declare that they have no conflicts of interest.

## CONTRIBUTIONS

PHN: Conseptualization, analysis, draft manuscript, consolidation of co‐athor comments, revisions, and paper finalization. CS: Literature review, data interpretation, draft manuscript of select sections, revisions and paper finalization. AP and SK: Field work coordination, data analyses, draft manuscript of select sections, and manuscript review. LMT: Data analyses, data visualisation, and manuscript review. DA, MA, SI, KK and JE‐A: Data interpretation, and manuscript review and edits. PM: Review of statistical analyses, data interpretation, and manuscript review and edits. All authors read and approved the final submitted manuscript.

## Supporting information

**Table S1:** Participant flow for health providers, pregnant women and mothers of children <2y
**Table S2:** Background characteristics^1^ of the study sample participated both surveys before and during the COVID pandemic (February 2020 and September 2020)

## Data Availability

The data that support the findings of this study are available in the tables/figures and supplementary material of this article. Additional data are available upon request.
