# Supplementary material for: Provision and utilisation of health and nutrition services during COVID‐19 pandemic in urban Bangladesh
Source: Matern Child Nutr. 2021 Jul 15;17(4):e13218. doi: 10.1111/mcn.13218 (PMC8420106; doi:10.1111/mcn.13218)
Supplement: Supplementary file 1 — Table S1: Participant flow for health providers, pregnant women and mothers of children <2y Table S2: Background characteristics1 of the study sample participated both surveys before and during the COVID pandemic (February 2020 and September 2020) [file MCN-17-e13218-s001.docx]

**Supplementary Table 1: Participant flow**

|  | **Health providers** | **Pregnant women** | **Mothers with children < 2y** |
| --- | --- | --- | --- |
| **In-person interview in February 2020** | **59** | **498** | **528** |
| Did not agree to give interview | 2 | 3 | 13 |
| Did not pick up phone | 0 | 1 | 22 |
| Wrong phone number | 0 | 2 | 12 |
| Phone switched off | 0 | 17 | 84 |
| Respondent refused to continue with survey | 0 | 3 | 5 |
| Delivered the child | NA | 418 | NA |
| Miscarriage/Abortion | NA | 8 | NA |
| Others (specify) | 0 | NA | 5 |
| Quit job, currently | 7 | NA | NA |
| Participated in pre-test | 5 | 6 | 0 |
| **Interview completed** | **45** | **40** | **387** |

NA: Not available

**Supplementary Table 2: Background characteristics^1^ of the study sample participated both surveys before and during the COVID pandemic (February 2020 and September 2020)**

|  | **Health providers** | **Pregnant women** | **Mothers** |
| --- | --- | --- | --- |
|  | **(n = 45)** | **(n= 40)** | **(n= 387)** |
| Age of respondents (years) | 34.6 ± 10.4 | 23.3 ± 4.4 | 25.2 ± 5.1 |
| Years of working tin the facility | 4.8 ± 3.9 | NA | NA |
| Designation |  | NA | NA |
| Physician/Medical officer | 22.2 | NA | NA |
| Nurse | 4.44 | NA | NA |
| Counsellor | 11.1 | NA | NA |
| Community worker | 6.67 | NA | NA |
| Paramedic | 48.9 | NA | NA |
| Medical Assistant | 4.44 | NA | NA |
| Nutritionist | 2.22 | NA | NA |
| Access to own smartphone with internet | 71.1 | NA | NA |
| Gestational age | NA | 36.5 ± 2.6 | NA |
| Education (years) | NA | 9.0 ± 3.3 | 9.6 ± 4.0 |
| No schooling | 0.0 | 2.5 | 2.8 |
| Primary school (grade1-5) | 0.0 | 35.0 | 35.7 |
| Middle school (grade 6-9) | 6.7 | 37.5 | 22.0 |
| High school (grade 10-12) | 26.7 | 17.5 | 16.3 |
| College or higher | 66.7 | 7.5 | 23.3 |
| Main occupation |  |  |  |
| Housewife | NA | 90.0 | 90.2 |
| Others | NA | 10.0 | 9.8 |
| Child age, mos | NA | NA | 10.0 ± 2.8 |
| Child sex (male) | NA | NA | 50.9 |
| Number of living children | NA | 1.2 ± 0.4 | 1.6 ± 0.8 |
| Religion |  |  |  |
| Muslim | 75.6 | 97.5 | 95.9 |
| Hindu | 13.3 | 2.5 | 4.1 |
| Christian | 11.1 | 0.0 | 0.0 |
| Household socio-economic status |  |  |  |
| Quintile 1 | NA | 25.0 | 31.8 |
| Quintile 2 | NA | 37.5 | 31.5 |
| Quintile 3 | NA | 37.5 | 36.7 |

^1^Background data presented in this table were from in -person survey in February 2020. NA: Not available
